# Fractional flow reserve (FFR) versus angiography in guiding management to optimise outcomes in non-ST segment elevation myocardial infarction (FAMOUS-NSTEMI) developmental trial: cost-effectiveness using a mixed trial- and model-based methods

**DOI:** 10.1186/s12962-015-0045-9

**Published:** 2015-11-14

**Authors:** Julian Nam, Andrew Briggs, Jamie Layland, Keith G. Oldroyd, Nick Curzen, Arvind Sood, Kanarath Balachandran, Raj Das, Shahid Junejo, Hany Eteiba, Mark C. Petrie, Mitchell Lindsay, Stuart Watkins, Simon Corbett, Brian O’Rourke, Anna O’Donnell, Andrew Stewart, Andrew Hannah, Alex McConnachie, Robert Henderson, Colin Berry

**Affiliations:** Health Economics and Health Technology Assessment, University of Glasgow, Glasgow, UK; Institute of Cardiovascular and Medical Sciences, BHF Glasgow Cardiovascular Research Centre, University of Glasgow, Glasgow, UK; Department of Cardiology, Golden Jubilee National Hospital, Agamemnon Street, Clydebank, G81 4DY UK; Department of Cardiology, University Hospital Southampton Foundation Trust, Southampton, UK; Department of Cardiology, Hairmyres Hospital, East Kilbride, UK; Department of Cardiology, Royal Blackburn Hospital, Blackburn, UK; Department of Cardiology, Freeman Hospital, Newcastle, UK; Department of Cardiology, City Hospitals Sunderland Foundation Trust, Sunderland, UK; Department of Cardiology, Aberdeen Royal Infirmary, Aberdeen, UK; Robertson Centre for Biostatistics, University of Glasgow, Glasgow, UK; Department of Cardiology, Nottingham University Hospitals NHS Trust, Nottingham, UK

## Abstract

**Background:**

In the Fractional flow reserve (FFR) versus angiography in guiding management to optimise outcomes in non-ST elevation myocardial infarction (FAMOUS) clinical trial, FFR was shown to significantly reduce coronary revascularisation, compared to visual interpretation of standard coronary angiography without FFR. We estimated the cost-effectiveness from a UK National Health Service perspective, based on the results of FAMOUS.

**Methods:**

A mixed trial- and model-based approach using decision and statistical modelling was used. Within-trial (1-year) costs and QALYs were assembled at the individual level and then modelled on subsequent management strategy [coronary artery bypass graft (CABG), percutaneous coronary intervention (PCI) or medical therapy (MT)] and major adverse coronary events (death, MI, stroke and revascularisation). One-year resource uses included: material, hospitalisation, medical, health professional service use and events. Utilities were derived from individual EQ5D responses. Unit costs were derived from the literature. Outcomes were extended to a lifetime on the basis of MACE during the 1st year. Costs and QALYs were modelled using generalized linear models whilst MACE was modelled using logistic regression. The analysis adopted a payer perspective. Costs and outcomes were discounted at 3.5 %.

**Results:**

Costs were related to the subsequent management strategy and MACE whilst QALYs were not. FFR led to a modest cost increase, albeit an imprecise increase, over both the trial [£112 (−£129 to £357)] and lifetime horizons [£133 (−£199 to £499)]. FFR led to a small, albeit imprecise, increase in QALYs over both the trial [0.02 (−0.03 to 0.06)] and lifetime horizons [0.03 (−0.21 to 0.28)]. The mean ICER was £7516/QALY and £4290/QALY over the trial and lifetime horizons, respectively. Decision remained high; FFR had 64 and 59 % probability of cost-effectiveness over trial and lifetime horizons, respectively.

**Conclusions:**

FFR was cost-effective at the mean, albeit with considerable decision uncertainty. Uncertainty can be reduced with more information on long-term health events.

**Electronic supplementary material:**

The online version of this article (doi:10.1186/s12962-015-0045-9) contains supplementary material, which is available to authorized users.

## Background

Non-ST segment elevation myocardial infarction (NSTEMI) is the most common form of acute coronary syndromes (ACS) [1]. The decision for coronary revascularisation is currently guided by visual interpretation of a coronary angiogram [1–3]. Visual interpretation, however, is subjective, potentially inaccurate and a cause for misdiagnosis and incorrect treatment decisions [4–6].

Myocardial FFR (FFRmyo) is defined as the maximal blood flow to the subtended myocardium in the presence of a stenosis, compared to maximum flow in the absence of a stenosis. An FFR ≤ 0.80 is correlated with the presence of inducible ischemia whereas an FFR ≥ 0.80 indicates patients can be managed safely with medical therapy (MT) [7]. Recent studies have demonstrated the value of fractional flow reserve (FFR) in guiding treatment decisions [8–13]. The actual impact of FFR on prospective management strategies has only recently been explored [10]. In addition, it is in patients with unstable coronary artery disease, particularly NSTEMI, who are managed by an invasive treatment strategy where FFR may prove to be clinically most useful.

The Fractional flow reserve versus Angiography in guiding Management to Optimise oUtcomeS in Non-ST-segment Elevation Myocardial Infarction (FAMOUS—NSTEMI, here referred to as ‘FAMOUS’) (NCT02073422) was a randomised multicentre pilot trial designed to evaluate impact of FFR vs. coronary angiography without FFR on subsequent management strategy [14] including either percutaneous coronary intervention (PCI), coronary artery bypass graft (CABG) or MT. FAMOUS had a follow-up of 1 year. When FFR results were disclosed in the FFR-guided group, the management strategy changed in 21.6 % of patients and resulted in a higher proportion of management with MT compared to the coronary angiography-guided group.

As FFR is a diagnostic test, the true value of FFR relates to its impact on patient management and outcomes [15–17]. This study focuses on the outcomes following patient management both within and beyond the trial time period. The aim of this study was to evaluate the cost-effectiveness of FFR compared with standard coronary angiography in patients with NSTEMI.

## Methods

This study expands on the 1-year clinical results of FAMOUS [14] by considering the cost-effectiveness. We used a mixed model- and trial-based approach with decision and statistical modelling. Decision modelling can present patient outcomes as part of the clinical pathway [18, 19]; statistical modelling can identify and reduce heterogeneity [20]. This can be especially important if trial endpoints are then used to estimate final economic endpoints.

The base population was patients with recent NSTEMI (mean 62 years old). The comparators were FFR-guided management and standard angiography-guided management. Economic outcomes were costs and quality-adjusted life-years (QALYs). Outcomes were viewed from a single health payer perspective—namely, the National Health Service (NHS). The analysis was conducted for both the 1-year trial time horizon as well as a lifetime horizon.

### Model

**Fig. 1:**
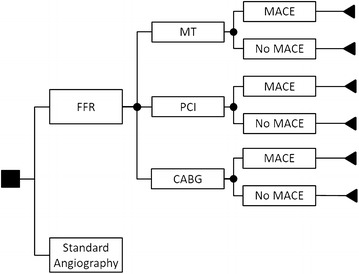
Model structure showing subsequent management strategy and MACE. Model structure is the same for angiography

The decision problem is summarised by the following decision analytic model, which was adapted from a previous design [21] (Fig. 1). Health and cost consequences were modelled on the treatment decision (MT, PCI or CABG) because treatment decision was directly informed by FFR or standard angiography. Outcomes were modelled additionally on incident major adverse cardiovascular outcomes (MACE) (death, myocardial infarction, stroke and revascularisation) because MACE served as the intermediate endpoint to model life expectancy beyond the trial time horizon, a common practice in previous models [2, 21]. Following the index year, a common QALY and cost tariff was applied to all years, dependent on the presence of MACE during the index year.

### Parameter sources

The probability of MT, PCI or CABG as well as the probability of MACE following each respectively was derived from the FAMOUS study. The life expectancy beyond 1 year following MACE was derived from a previous model in patients with unstable angina or NSTEMI [2, 21].

Trial period resource use was obtained from FAMOUS and unit costs were obtained from the literature. Individual level costs were assembled by multiplying resource use with unit costs. Resource use included: the pressure wire intervention; the treatment strategy (PCI, CABG or medical management); catheterisation laboratory time; Coronary Care Unit (CCU), Intensive Treatment Unit (ITU) and general ward days; other in-hospital procedures (x-rays and echocardiograms) and health events (rehospitalisation, revascularisation, myocardial infarction, and stroke). Regarding the treatment strategy, the analysis included use of PCI materials (catheters, balloons and stents) and drugs (glycoprotein IIa/IIIb inhibitors, bivalirudin, clopidogrel). The use of a pressure wire in patients randomised to coronary angiography alone was removed from the cost estimates as it was protocol driven. Unit cost information was generally derived from national sources, including NHS Reference Costs, the British National Formulary (http://www.bnf.org), Information Services Division Scotland [22] and NICE Clinical Guidelines [2]. Unit cost parameter information is presented in Table 1.

**Table 1:** Data inputs used in the model

	Mean	SE	Distribution	Source
Treatment decision probabilities				
MT, FFR	0.23	0.03	Dirichlet	FAMOUS
PCI, FFR	0.71	0.02	Dirichlet	FAMOUS
CABG, FFR	0.06	0.02	Dirichlet	FAMOUS
MT, standard care	0.13	0.02	Dirichlet	FAMOUS
PCI, standard care	0.80	0.01	Dirichlet	FAMOUS
CABG, standard care	0.07	0.02	Dirichlet	FAMOUS
Equipment costs (£):				
Guiding catheter	20	0	–	National procurement
Guidewire	20	0	–	National procurement
Pressure wire	270	0	–	National procurement
Adenosine vial	12	0	–	BNF
Balloon catheter	50	0	–	National procurement
Drug eluting stent	290	0	–	National procurement
Bare metal stent	90	0	–	National procurement
Tirofiban (avg/patient)	242	69	Gamma	BNF; evidence.nhs.uk
Bivalirudin (avg/patient)	625	48	Gamma	BNF; evidence.nhs.uk
Clopidogrel (per month)	2		–	BNF; evidence.nhs.uk
Procedure costs (£):				
CABG	5041	313	Gamma	NHS reference costs
Echocardiogram	128	26	Gamma	Golden Jubilee National Hospital
Optical coherence tomography	1020	204	Gamma	Golden Jubilee National Hospital
Intravascular ultrasound	540	108	Gamma	Golden Jubilee National Hospital
Chest x-ray	18	4	Gamma	Golden Jubilee National Hospital
Hospitalisation costs (£):				
Cath lab time (per hour)	1681	301	Gamma	ISD Scotland
Day in CCU	1492	60	Gamma	Golden Jubilee National Hospital
Day in ITU	2288	458	Gamma	Golden Jubilee National Hospital
Day in general ward	303	17	Gamma	NHS reference costs
Inhospital event costs (£):				
Severe bleeding	222	3	Gamma	NHS reference costs
Stroke	2709	129	Gamma	NHS reference costs
MI	1492	76	Gamma	NHS reference costs
Event costs (£):				
Rehospitalisation	2261	452	Gamma	NICE CG94
Revascularisation	2477	63	Gamma	NHS reference costs
MI	2261	452	Gamma	NICE CG94
Disease related costs (index year) (£):				
Index year after additional MI	3228	215	Gamma	NICE CG94
Index year of stroke	16,926	428	Gamma	NICE CG68
Additional life-expectancies (years)				
MACE	10.70	2.20	Normal	Kent et al., NICE CG94
No MACE	5.96	1.30	Normal	Kent et al., NICE CG94
Other long-term parameters				
Annual cost post year 1 (£)	423	55	Gamma	Kent et al., NHS reference costs
MACE utility change	−0.05	0.04	Beta	Kent et al., Palmer et al.

Long-term annual disease costs for those with and without MACE were derived from a previous model of FFR in NSTEMI patients [21].

Trial period QALYs were obtained from FAMOUS by integrating the area under the curve of health utilities. Health utilities at presentation, 6 months and 1 year were estimated with the EuroQol 5D-3L instrument. Responses were converted into utilities with the use of a UK-specific algorithm [23]. For the long-term extension, the utility of those without MACE followed the general population and declined with increasing age in the model. The mean utility change (decrement) of those with MACE was estimated to be −0.05 [21, 24].

### Analysis

We planned to model one-year trial costs and QALYs on the treatment decision (MT, PCI or CABG) and incident MACE. To estimate the cost parameters, we derived adjusted cost estimates fitting a generalized linear model (GLM) and performing marginal prediction. GLM is the appropriate method when the objective is to obtain efficient estimation of skewed variables [20, 25–27]. Appropriate family and link functions were determined using the Modified Park’s and Hosmer and Lemeshow tests, respectively. The regression included a treatment term in addition to other relevant baseline characteristics, selected by a combination of clinical reasoning, independent association with the dependent variable at the p < 0.25 level and prevalence >10 %. The final form of the cost model included the following additional covariates: utility at presentation, age, sex, smoking status and history of PCI. The final form of the QALYs model included the following additional covariates: utility at presentation, age, sex and smoking status.

Estimation of MACE following treatment decision (MT, PCI or CABG) was derived in a similar manner. The final form of the model included the following covariates: utility at presentation, age, sex and history of chronic obstructive pulmonary disease. A logistic regression was used to regress MACE on treatment decision, along with baseline characteristics. The ORs for PCI and CABG were then extracted and applied to a baseline MT, which was estimated using marginal prediction. ORs were applied to odds [risk/(1-risk)] and converted to risks (odds/(1 + odds)) in the model.

We first tested the significance of the model structure using analysis of variance tests. Partial analysis of variance tests were conducted using deviances from an ANOVA and a *p* value using the Chi-Squared distribution. If the model structure in Fig. 1 showed no incremental value, raw trial estimates were used.

The developed statistical models were then used to estimate outcomes using marginal prediction. Any observed comparison of two groups, whether randomised or not, is likely to show imbalance in baseline covariates, regardless of statistical significance. Marginal prediction effectively holds all else equal, save for the predictor of interest—treatment.

Missing data was imputed using multiple imputation with chained equations (MICE), where appropriate [28].

We used bootstrapping and probabilistic sensitivity analysis to incorporate sampling uncertainty, parameter uncertainty and model uncertainty. Beta, gamma and lognormal distributions were generally used for utility, cost and relative risk parameters. A dirichlet distribution was used to randomly sample >2 rival events, such as the treatment management decision of MT, PCI or CABG. We conducted model selection in Stata 12, statistical modelling in R 3.2 [29] and decision analytic modelling in Microsoft Excel. All costs are presented in 2014 British Pound Sterling.

### Research ethics

The trial was approved by the National Research Ethics Service (reference 11/S0703/6) and complies with the Declaration of Helsinki. The study information sheet that had been approved by the research ethics committee was provided to each participant. Written informed consent was obtained before the diagnostic coronary angiogram and randomization.

## Results

### Unadjusted trial outcomes

EQ-5D-3L responses were missing in 17 and 24 % of the trial population at 6- and 12-months, respectively. However, baseline characteristics were balanced across missing and complete groups (Additional file 1); as well, missingness was not statistically associated with intervention (Chi-squared test; χ^2^ = 0.0234; df = 1, p = 0.88). Missing at random was thus a plausible conclusion and multiple imputation was subsequently conducted (Additional file 1).

Following FFR, patients received MT, PCI and CABG with probabilities of 23, 71 and 6 %, respectively. Following standard angiography, patients received MT, PCI and CABG with probabilities of 13, 80 and 7 %, respectively.

Utilities and resource use—unadjusted—for the trial population are tabulated in the Additional file 1. Compared to the standard care group, the utilities in the FFR group were lower at presentation (0.78 vs 0.80) but comparable by 6-months (0.83 vs 0.83) and higher at 12-months of follow-up (0.83 vs. 0.80), although none of the differences were significant (Additional file 1).

FFR measurement required an average of 1.03 pressure wires. Lower revascularisation meant lower use of PCI materials such as balloon catheters, drug eluting stents and bare metal stents. Following management with FFR, use of echocardiography was higher whilst OCT and x-rays were no different to standard care. Patients managed with FFR passed similar amounts of time in the catheter laboratory but less ITU time and general ward time. For incident events following treatment, the FFR group had slightly lower rates of rehospitalisation and MI whilst having similar rates of revascularisation, stroke and death compared to standard care; however event rates were extremely low in some and none displayed statistical significance (Additional file 1).

Unadjusted, raw mean costs and QALYs are presented in Table 2. FFR measurement led to increased costs of pressure wire use (+£279) and cath lab time (+£57) which was offset modestly by savings in PCI (−£92), CABG (−£32) and medication use (−£17). However, the largest cost savings following FFR were in reduced hospital length of stay (−£331) and index year events (−£243) but they also displayed the greatest uncertainty. Overall, there was a mean cost saving of £349 and an incremental 0.02 QALYs following FFR during the 1st year.

**Table 2:** Raw unadjusted total and incremental costs and QALYs following standard care and FFR management

	Standard care	FFR	Difference
	Mean (95 % CI)	Mean (95 % CI)	Mean (95 % CI)
Cost (£)			
Pressure wire	0 (0–0)	279 (273–287)	279 (273–287)
PCI	837 (750–932)	766 (689–844)	−72 (−193 to 45)
CABG	346 (174–550)	314 (143–487)	−32 (−292 to 226)
Medications	59 (48–71)	49 (38–60)	−17 (−42 to 7)
Cath lab time	1806 (1672–1949)	1864 (1770–1962)	57 (−114 to 221)
Length of stay	4435 (4043–4847)	4104 (3595–4673)	−331 (−1002 to 356)
Other procedures	125 (117–132)	134 (127–141)	9 (−1 to 20)
Events during index year	956 (613–1372)	713 (468–982)	−243 (−736 to 200)
Total cost (£)	8565 (7872–9304)	8222 (7518–8985)	−349 (−1367 to 675)
Total QALYs	0.801 (0.765–0.835)	0.82 (0.787–0.845)	0.02 (−0.029 to 0.061)

### Statistical models

Results of the statistical models are provided in the Additional file 1. Modelling cost on the treatment decision interacted with MACE and displayed statistical significance under a partial analysis of deviance test (p < 0.001); modelling MACE on the treatment decision trended towards statistical significance (p = 0.07). For QALYs, however, the addition of treatment decision interacted with MACE did not improve the model (p = 0.37). The model structure in Fig. 1 was thus used for the estimation of cost and MACE whilst it was not imposed for QALYs; instead, unadjusted raw QALYs were used for the trial period.

Table 3 presents the adjusted estimates from the statistical models. As expected, 1-year costs were higher in the presence of MACE vs. no MACE. MT represented the lowest cost, followed by PCI and CABG. Table 3 also presents the extracted odds-ratios of MACE for PCI and CABG and the marginal predicted probability of MACE following MT. Compared to MT, PCI did not increase the odds of MACE appreciably, whilst CABG increased the odds over threefold.

**Table 3:** Statistical model outputs used in the decision analytic model

	Mean	SE
Decision model costs (£)		
MT, MACE	9622	864
PCI, MACE	14,894	589
CABG, MACE	21,851	1984
MT, no MACE	5819	139
PCI, no MACE	7204	72
CABG, no MACE	17,774	482
MACE parameters		
MT (probability)	0.09	0.01
OR for PCI	1.01	(0.35–2.86)^a^
OR for CABG	3.80	(0.98–14.62)^a^

^a^95 % confidence interval

### Cost-effectiveness

Mean results of the cost-effectiveness analysis are presented for both the one-year trial and lifetime time horizons (Table 4). FFR led to a mean additional £112 and 0.01 QALYs over the trial time horizon, compared to standard angiography, whilst over a lifetime it led to an additional £133 and 0.03 QALYs. The mean ICERs were £7516/QALY and £4290/QALY over the trial and lifetime horizons, respectively.

**Table 4:** Results of cost-effectiveness by trial (1-year) and lifetime time horizons. Mean and 95 % CI presented

	Standard		FFR		Incremental		ICER
	Costs (£)	QALYs	Costs (£)	QALYs	Costs (£)	QALYs	(£/QALY)
Trial	7574 (6963–8443)	0.80 (0.76–0.84)	7686 (7141–8482)	0.82 (0.79–0.85)	112 (−129 to 357)	0.01 (−0.03 to 0.06)	7516
Lifetime	10,954 (9482–12,614)	6.30 (5.07–7.23)	11,087 (9652–12,699)	6.33 (5.09–7.25)	133 (−199 to 499)	0.03 (−0.21 to 0.28)	4290

**Fig. 2:**
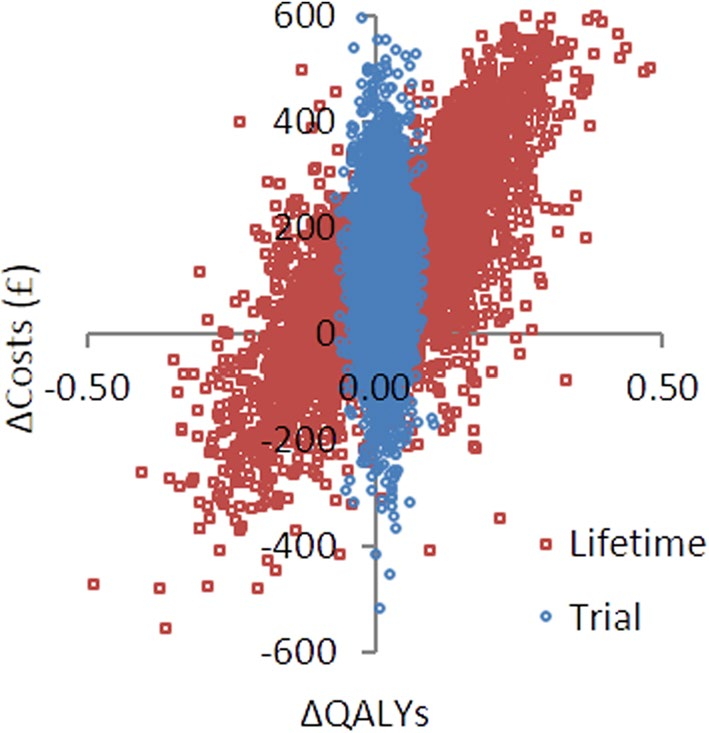
Cost-effectiveness plane displaying incremental costs vs. incremental QALYs

**Fig. 3:**
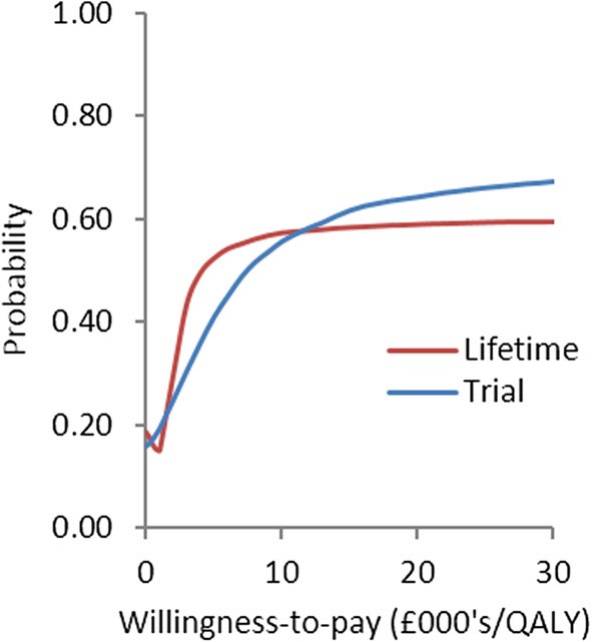
Cost-effectiveness acceptability *curves* for the trial and lifetime time horizons

The cost-effectiveness plane displays the variability in incremental costs and QALYs (Fig. 2). Variability in both incremental costs and QALYs was considerably greater under the lifetime horizon, compared to the trial time horizon. Both time horizons presented consistent results of increased costs against a background of comparable QALYs. This relative comparability in incremental QALYs presents itself more clearly in the cost-effectiveness acceptability curve (CEAC) (Fig. 3). The cost-effectiveness of FFR remained relatively uncertain throughout the willingness-to-pay (WTP) range of £20,000–£30,000/QALY for both the trial time horizon (64–67 %) and the lifetime horizon (~59 %) (Fig. 3).

## Discussion

Our analysis presents an early estimate of the combined health and economic impact of FFR-guided management in patients with NSTEMI as measured by FAMOUS. Both the trial and lifetime horizons present consistent results of a modest, relatively precise incremental cost (£112 and £133, respectively) along with modest, albeit imprecise, incremental QALYs (0.01 and 0.03, respectively). FFR displays an acceptable ICER of £4290/QALY. However, decision uncertainty remains considerable.

The strength of the present analysis is its use of trial data to reduce heterogeneity in economic outcomes through the use of statistical modelling and marginal prediction. Statistical modelling identified the sources of heterogeneity and distilled the attributable effect of the treatment management (MT, PCI and CABG) along with MACE; one related to the critical decision in the patient management pathway whilst the other related to the critical patient outcomes. Marginal prediction improved estimation of economic outcomes by reducing heterogeneity unrelated to the patient management pathway and critical patient outcomes.

The present results highlight the impact of modelling for within-trial assessments. The impact statistical modelling and marginal prediction on the variability was apparent if one compares the variability of trial period costs in the raw vs. modelled results. FFR displayed raw cost-savings during the trial time horizon. Results, however, were very imprecise around a zero difference. By contrast, the decision analysis reduced the 95 % CI nearly six-fold and distilled a more clear direction of the distribution. These adjusted results suggested more modest downstream cost savings that “recouped” part, but not all, of the intervention cost, leading to an overall modest cost increase. As clinical trials becoming an increasingly common source of evidence to assess the value for money of health interventions, analysts should consider such modelling practices.

Downstream cost savings were driven chiefly by absolute, though non-significant, reductions in length of stay and health events such as revascularizations, rehospitalisations, MI and stroke. An improvement of the present analysis would be to model each important outcome separately, rather than as a composite. Composite outcomes make it difficult to attribute the appropriate individual cost and health-related utility tariffs. However, low event counts precluded a more detailed decision model that could characterise health events separately with statistical modelling and marginal prediction.

Inadequate information size was the greatest limitation of the FAMOUS pilot to inform cost-effectiveness. The primary objective of FAMOUS was to gather evidence on the FFR strategy in NSTEMI patients, and although the trial was designed to measure health outcomes at 1 year, it was not powered for these events. The resulting uncertainty was apparent in costs of length of stay and events during the index year, with confidence intervals at least two/threefold greater than other cost categories. Low event counts precluded a more detailed decision model that could characterise downstream MI, rehospitalisation, and other events as functions of the patient management pathway. Low information size also precludes a strong method to extend trial outcomes to a lifetime using the observed data. In order to estimate the wider lifetime impact of FFR, economic modelling would generally extend trial outcomes. This is generally done by extrapolating survival and weighting it with an appropriate quality-of-life utility. However, FAMOUS pilot measured only two time points and observed 8 deaths making any extrapolation uninformative.

The strength of the analysis is also a potential limitation. The decision analysis necessarily applies a structure to the decision problem. We believe the present structure to best represent the patient management pathway and the downstream important patient outcomes. The model structure attributes differences through these pathways. However, it may be the case that differences occur through an alternative pathway. Modelling, however, is unavoidable when extending outcomes beyond the trial horizon. Given the low information size and limited follow-up, the long-term model conditioned life expectancy on MACE. A common utility decrement was applied; however, those with MACE during the index year may return to quality of life similar to those who didn’t have MACE, at some point in the future. This would likely increase decision uncertainty by depressing the CEAC further towards 50 %. There may be differences in health events between FFR and standard angiography-led management that do not present until well after the index year. At present, there is no indication of this but a larger planned future trial will measure this.

There is only one known study comparing economic outcomes for patients with NSTEMI, by Kent et al. [21]. While their estimates of treatment following FFR were based on data of hypothetical treatment decisions, the present study uses actual decision data from the FAMOUS trial. The hypothetical results underestimated the actual reduction in primary revascularization following FFR (OR 0.88 vs 0.52, using raw unadjusted trial results). We found comparable incremental QALYs during the index year to the Kent et al. study. We also found FFR led to cost-savings in similar areas, namely index treatment costs and downstream health event costs.

Consistently low mean ICERs over short- and long-term horizons suggest FFR may represent a cost-effective resource allocation over standard angiography. The cost-effectiveness of FFR, however, is met with considerable decision uncertainty. Uncertainty can be reduced with more information on long-term health events. Clinical trials are now a common source of evidence to assess the value for money of health interventions. Analysts should consider the benefits of a mixed model- and trial based-approach (or analysis) with decision and statistical modelling.

## Conclusions

FFR-guided management of NSTEMI may be a cost-effective strategy over standard angiography, showing that more targeted invasive management can reduce healthcare resource costs without compromising patient outcomes. However, there still remains considerable decision uncertainty which can be reduced with increased information size and additional long-term evidence on major adverse cardiac events.

## Additional file

10.1186/s12962-015-0045-9 Resource use.
