# Supplementary material for: Fractional flow reserve (FFR) versus angiography in guiding management to optimise outcomes in non-ST segment elevation myocardial infarction (FAMOUS-NSTEMI) developmental trial: cost-effectiveness using a mixed trial- and model-based methods
Source: Cost Eff Resour Alloc. 2015 Nov 14;13:19. doi: 10.1186/s12962-015-0045-9 (PMC4647286; doi:10.1186/s12962-015-0045-9)
Supplement: Supplementary file 1 — 10.1186/s12962-015-0045-9 Resource use. [file 12962_2015_45_MOESM1_ESM.docx]

**Resource use**

*Table 1. Resource use across materials, procedures, hospitalisation and events.*

|  | Standard Care | FFR | Δ | p |
| --- | --- | --- | --- | --- |
|  | *Mean (SD)* | *Mean (SD)* | *Mean* |  |
| *Diagnosis/treatment guidance* |  |  |  |  |
| Pressure wires | 0.00 (0.00) | 1.03 (0.18) | 1.03 | <0.01 |
| Coronary guidewires | 1.79 (1.06) | 0.59 (0.77) | -1.20 | <0.01 |
| Guide catheters | 1.43 (0.63) | 1.42 (0.59) | 0.00 | 0.85 |
| *Revascularisation* |  |  |  |  |
| - CABG (% [#]) | 7 [12] | 6 [11] | -1 | 0.83 |
| - MT (% [#]) | 13 [23] | 23 [40] | 10 | 0.03 |
| - PCI (% [#]) | 80 [139] | 71 [125] | -9 | 0.06 |
| Balloon catheters | 1.73 (1.67) | 1.40 (1.32) | -0.33 | 0.09 |
| Drug eluting stents | 1.13 (1.22) | 0.96 (1.08) | -0.17 | 0.23 |
| Bare metal stents | 0.28 (0.79) | 0.19 (0.54) | -0.09 | 0.60 |
| Adenosine vials | 28.47 (32.26) | 28.82 (27.83) | 0.35 | 0.65 |
| GP IIb/IIIa use (% [#]) | 26 [45] | 19 [34] | -7 | 0.16 |
| Bivalirudin use (% [#]) | 1 [2] | 1 [2] | 0 | 1.00 |
| *Procedures* |  |  |  |  |
| IVUS (% [#]) | 6 [10] | 3 [6] | -2 | 0.32 |
| OCT (% [#]) | 0 [0] | 0 [0] | 0 | 1.00 |
| ECHO (% [#]) | 83 [145] | 89 [157] | 6 | 0.12 |
| X-Ray (% [#]) | 93 [161] | 94 [165] | 1 | 0.68 |
| *Hospitalisation* |  |  |  |  |
| Cath lab hours | 1.07 (0.56) | 1.11 (0.39) | 0.03 | <0.01 |
| CCU days | 1.94 (1.96) | 1.72 (2.26) | -0.22 | 0.10 |
| ITU days | 0.01 (0.11) | 0.05 (0.33) | 0.04 | 0.25 |
| Gen. Ward Days | 4.99 (3.92) | 4.68 (3.67) | -0.31 | 0.52 |
| *Incident events following treatment* |  |  |  |  |
| Rehospitalisation (% [#]) | 22 [38] | 19 [34] | -3 | 0.60 |
| Revascularisation (% [#]) | 5 [8] | 5 [8] | 0 | 1.00 |
| MI (% [#]) | 5 [8] | 3 [5] | -2 | 0.41 |
| Stroke (% [#]) | 1 [2] | 0 [0] | -1 | 0.25 |
| Death (% [#]) | 2 [4] | 3 [5] | 1 | 1.00 |

Table 2. Trial utilities from EQ-5D responses.

|  | Standard Care | FFR | Δ | p |
| --- | --- | --- | --- | --- |
|  | Mean (SD) | Mean (SD) | Mean |  |
| Utility at presentation | 0.80 (0.24) | 0.78 (0.28) | -0.02 | 0.88 |
| Utility at 6-months | 0.83 (0.24) | 0.83 (0.23) | 0.00 | 0.89 |
| Utility at 12 months | 0.80 (0.27) | 0.83 (0.23) | 0.03 | 0.66 |

Statistical models

Table 3. GLM of total costs. Gamma family and identity link.

|  | Mean | SE | p-value |
| --- | --- | --- | --- |
| Intercept | 5314 | 1055 | p<0.001 |
| PCI | 1386 | 356 | p<0.001 |
| CABG | 11955 | 1682 | p<0.001 |
| MACE | 3803 | 1620 | 0.02 |
| PCI*MACE | 3886 | 2028 | 0.06 |
| CABG*MACE | 274 | 4129 | 0.94 |
| Utility at presentation | -364 | 642 | 0.57 |
| Age | 24 | 14 | 0.10 |
| Sex | -654 | 372 | 0.08 |
| Former smoker | -379 | 371 | 0.31 |
| Never smoked | 66 | 385 | 0.87 |
| History of PCI | -919 | 418 | 0.03 |

Table 4. Logistic regression of MACE.

|  | Mean | SE | p-value |
| --- | --- | --- | --- |
| Intercept | -1.88 | 1.28 | 0.14 |
| PCI | 0.01 | 0.53 | 0.99 |
| CABG | 1.33 | 0.69 | 0.05 |
| Utility at presentation | -1.66 | 0.60 | 0.01 |
| Age | 0.01 | 0.02 | 0.57 |
| Male | -0.10 | 0.42 | 0.82 |
| Constructive obstructive pulmonary disease | 1.02 | 0.49 | 0.04 |

**Missingness**

*Table 5. Baseline characteristics for patients with complete EQ-5D-3L information and those without 6- or 12-month information.*

|  | Complete | Missing |
| --- | --- | --- |
| n (%) | 235 (0.67) | 115 (33%) |
| Age (years) | 63.36 (11.18) | 59.08 (10.28) |
| Male (%) | 176 (0.75) | 84 (0.73) |
| Smoking (%) |  |  |
| Current | 92 (0.39) | 51 (0.44) |
| Former | 74 (0.31) | 28 (0.24) |
| Never | 69 (0.29) | 36 (0.31) |
| NYHA (%) |  |  |
| I | 204 (0.87) | 102 (0.89) |
| II | 23 (0.1) | 11 (0.1) |
| III | 4 (0.02) | 2 (0.02) |
| IV | 4 (0.02) | 0 (0) |
| CCS (%) |  |  |
| No symptoms | 0 (0) | 2 (0.02) |
| I | 1 (0) | 0 (0) |
| II | 2 (0.01) | 1 (0.01) |
| III | 17 (0.07) | 5 (0.04) |
| IV | 215 (0.91) | 107 (0.93) |
| Cardiac arrhythmia (%) | 17 (0.07) | 6 (0.05) |
| Hypercholesterol (%) | 86 (0.37) | 41 (0.36) |
| Hyptertension (%) | 109 (0.46) | 50 (0.43) |
| Renal Impairment | 13 (0.06) | 7 (0.06) |
| Serum Creatine (μmol/l) | 83.98 (48.8) | 86.86 (67.4) |
| eGFR (mL/min) | 96.32 (88.25) | 95.42 (75.98) |
| Coronary artery disease (%) | 120 (0.51) | 56 (0.49) |
| Diabetes (%) |  |  |
| No diabetes | 203 (0.86) | 95 (0.83) |
| Type I | 3 (0.01) | 2 (0.02) |
| Type II | 29 (0.12) | 18 (0.16) |
| Ischaemia | 56 (0.24) | 21 (0.18) |
| Previous angiogram (%) | 33 (0.14) | 17 (0.15) |
| Previous PCI (%) | 24 (0.1) | 14 (0.12) |
| Previous MI (%) | 31 (0.13) | 15 (0.13) |
| COPD (%) | 27 (0.11) | 12 (0.1) |
| PVD | 16 (0.07) | 12 (0.1) |
| TIA | 17 (0.07) | 7 (0.06) |
| Cancer | 15 (0.06) | 6 (0.05) |

Multiple imputation using chained equations

Multiple imputation was conducted using the MICE package in R 3.2. Parameters to be imputed included a number of outcomes at 6- and 12-months, including: MI, stroke, death, utility, NYHA status, angina CCS status, rehospitalisation and revascularisation. As well, for some patients, imputation of utility at presentation was required. Independent covariates included baseline characteristics (such as those in the preceding table) and upstream outcomes (aforementioned). Factor variables were imputed using polytomous regression while continuous variables were interpreted using predictive mean matching as recommended. We used 30 iterations and 5 imputations.
